# Case report: Three cases of systemic lupus erythematosus presenting primarily with massive ascites and significantly elevated CA-125 levels and a review of pseudo-pseudo Meigs’ syndrome in literature

**DOI:** 10.3389/fimmu.2024.1423631

**Published:** 2024-07-16

**Authors:** Qiyu Li, Bailing Tian

**Affiliations:** ^1^ Department of Cardiology, The First Hospital of China Medical University, Shenyang, Liaoning, China; ^2^ Department of Rheumatology and Immunology, The First Hospital of China Medical University, Shenyang, Liaoning, China

**Keywords:** SLE - systemic lupus erthematosus, CA12 - 5, Pseudo-pseudo Meigs' syndrome, ascite, multiple serous cavity effusion

## Abstract

This article presents three detailed case reports and a brief review of the literature on a rare manifestation of systemic lupus erythematosus (SLE) known as Pseudo-Pseudo Meigs' Syndrome (PPMS). The patients' condition was characterized by elevated CA-125 levels, massive ascites andpleural effusion which is typically associated with ovarian malignancies but can also present in various non-malignant conditions, including SLE. A thorough literature review was conducted, summarizing similar cases and their clinical outcomes to provide a broader understanding of this uncommon syndrome. The findings emphasize the need for heightened awareness and consideration of pseudo-pseudo Meigs' syndrome in patients with SLE presenting with unexplained ascites and pleural effusion.

## Introduction

We report three cases of systemic lupus erythematosus (SLE) that initially presented with significant pleural effusion, ascites, and marked elevation in Cancer Antigen 125 (CA-125) levels. After ruling out secondary causes such as tumors and infections, these cases were diagnosed as “pseudo-pseudo Meigs’ syndrome” (PPMS). In addition, we reviewed 30 cases of PPMS reported since 2005, summarizing the pathogenesis, clinical characteristics, and treatment strategies associated with this condition. This review provides new insights and perspectives on the clinical management and understanding of PPMS.

## Case presentation

**Figure 1:**
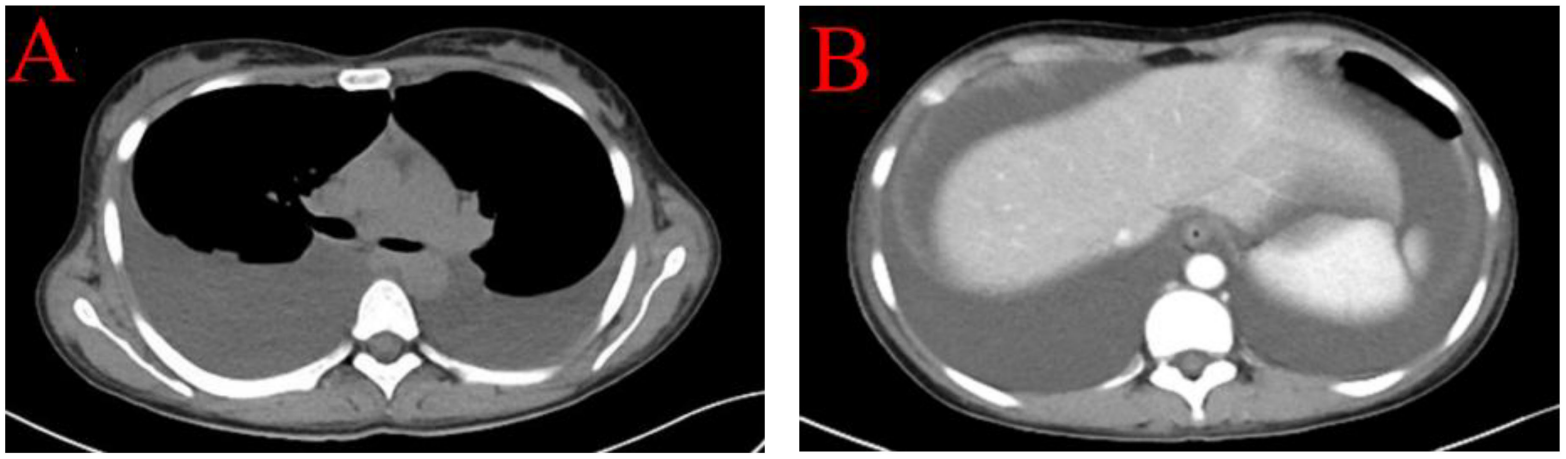
CT imaging revealed bilateral pleural effusions **(A)** and substantial abdominal ascites without signs of portal hypertension and space-occupying lesions **(B)**.

Case 1 involved a 28-year-old woman with no prior history of rheumatic or autoimmune diseases and an otherwise healthy medical background. Following an influenza infection two months earlier, she developed symptoms including dry mouth and facial edema, which progressed to scleral edema. Initial laboratory evaluations at a local hospital identified urine protein 1+, Anti-Ro-52 antibody (Ro52) +, Anti-Sjögren’s Syndrome Antigen A (SSA) +, Anti-Sjögren’s Syndrome Antigen B (SSB) +, albumin at 27.59 g/L, and normal creatinine levels. The initial treatment regimen included albumin supplementation and diuretics. However, her condition worsened over the next two weeks, characterized by generalized edema, fatigue, significant hair loss, and butterfly erythema on the cheeks, prompting further investigations. Subsequent tests showed positive antinuclear antibodies (ANA) with a nucleolar pattern at a titer of 1:1000, SSA 3+, SSB 3+, Ro-52 3+ and notably low complement levels (C3 0.33 g/L; C4 0.02 g/L), negative anti-dsDNA, albumin at 23.3 g/L, 24-hour urine protein quantification at 0.173 g, urine microalbumin at 163.00 mg/L, an echocardiogram indicating a small to moderate amount of pericardial effusion, normal renal function, and normal blood ions. Based on the 1997 American College of Rheumatology (ACR) (1) or 2012 Systemic Lupus Erythematosus International Collaborating Clinics criteria for the classification of SLE (2), the diagnosis of SLE was established, accompanied by an Systemic Lupus Erythematosus Disease Activity Index (SLEDAI) score of 11. Computed Tomography (CT) imaging revealed bilateral pleural effusions and substantial abdominal ascites without signs of portal hypertension (**Figure 1**), suggesting non-portal origins of ascites. Infectious causes were ruled out following thoracic and abdominal paracentesis and analysis of ascitic and pleural fluids. Ascitic fluid CA-125 levels were 858 U/mL, pleural fluid CA-125 levels were 325 U/mL, and serum CA-125 levels were 1225 U/mL. A comprehensive evaluation, including vaginal ultrasound and positron emission tomography-computed tomography (PET-CT), excluded malignancy. The patient was treated with intravenous methylprednisolone (MP) 250 mg for three days, followed by a tapered dose of oral prednisone 1 mg/kg/day, oral mycophenolate mofetil (MMF) 0.75 g twice daily, and subcutaneous Telitacicept 160 mg weekly (Telitacicept, a novel recombinant fusion protein consisting of Transmembrane Activator and CAML Interactor (TACI) and the Fc portion of human immunoglobulin G (IgG) (TACI-Ig), is designed to interfere with abnormal B cell and plasma cell activation by antagonizing the interaction of B Lymphocyte Stimulator (BlyS) and A Proliferation-Inducing Ligand (APRIL) with their receptors on B lymphocytes). Hydroxychloroquine (HCQ) 200 mg twice daily was discontinued due to intolerance. Remarkable improvement was observed after two weeks of therapy, leading to discharge. One month later, the patient had only residual ascites, resolved pleural effusion, normalized complement levels (C3 and C4), and reduced CA-125 levels to 98.5 U/mL.

Case 2 describes a 37-year-old woman who was diagnosed with Sjögren’s syndrome over a decade ago. She had been managing her condition with a daily regimen of 8 mg of MP and 200 mg of HCQ sulfate for the past two years, during which she reported good control of her symptoms without the need for additional intervention. Two months prior to presentation, she began experiencing abdominal distension and lower limb swelling, which she initially disregarded. Three weeks before seeking medical attention, following a Coronavirus disease 2019 (COVID-19), she noticed a significant reduction in urine output accompanied by pronounced shortness of breath, which prompted her to consult a healthcare professional.

**Figure 2:**
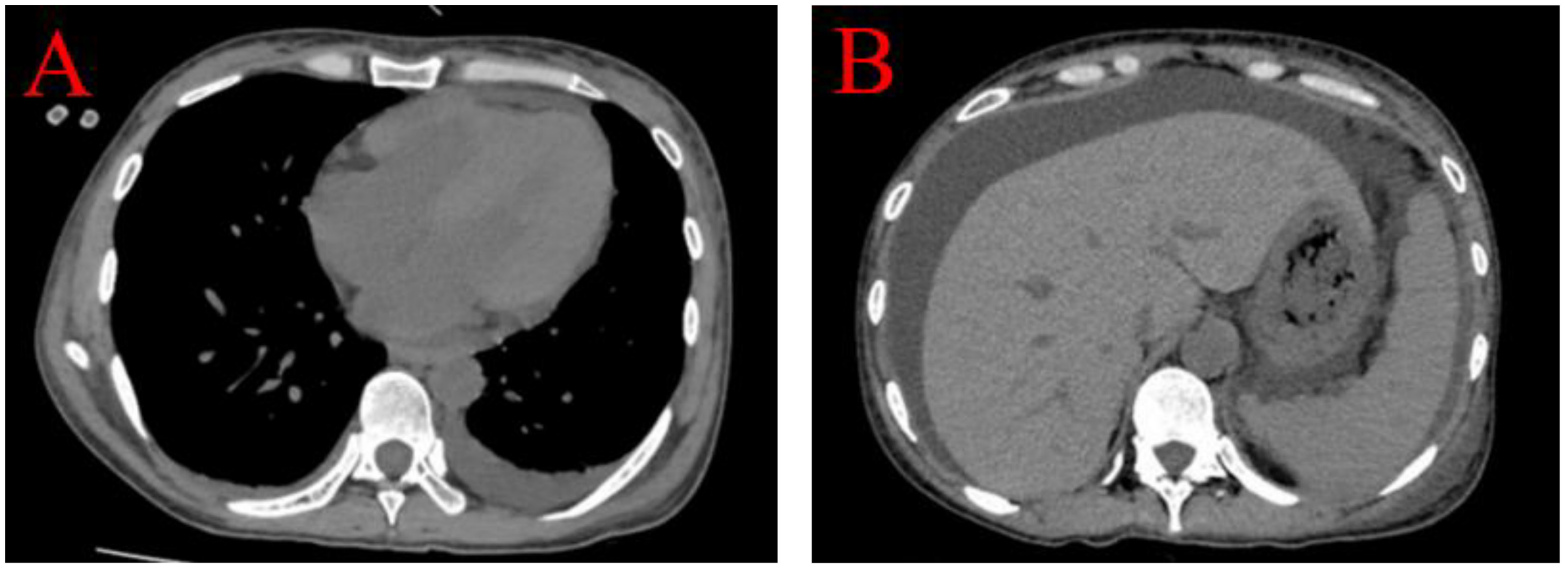
Bilateral pleural effusions were evident on pulmonary CT, predominant on the left side **(A)**. Abdominal CT scans revealed extensive ascites without portal hypertension and any space-occupying lesions **(B)**.

Upon evaluation, the patient exhibited marked abdominal distension. Abdominal ultrasound and CT scans revealed extensive ascites without any space-occupying lesions. Bilateral pleural effusions were evident on pulmonary CT (**Figure 2**), and transthoracic three-dimensional echocardiography revealed a small pericardial effusion. Laboratory tests showed positive ANA at a titer of 1:1000 with a nuclear speckled pattern, U1 Ribonucleoprotein (U1RNP) +, SSA+, RO-52+, anti-nucleosome antibody (ANUA) 2+, anti-histone antibody (AHA) 2+, anti-ribosomal P protein antibody (P) 3+, and anti-dsDNA (dsDNA) >800 IU/mL. Complement levels were significantly reduced (C3 0.33 g/L; C4 0.04 g/L), with elevated ferritin (541 µg/L) and CA-125 (186 U/mL). The patient presented with lupus nephritis, indicated by serum creatinine (SCr) of 402 µmol/L, urine protein 2+, a 24-hour urine protein quantity of 2.701 g, serum albumin 27.9 g/L, hemoglobin (HGB) 67 g/L, and platelets (PLT) 100 x 10^9/L (normal range 125-350).

Further assessment post-admission included abdominal paracentesis and drainage. Laboratory analyses showed normal white blood cell counts, negative adenosine deaminase (ADA), and negative acid-fast bacillus smear, allowing for the exclusion of infection and tuberculosis. Cytology was benign, ruling out malignancy, although ascitic fluid CA-125 levels were elevated at 359 U/mL. The patient was diagnosed with SLE and lupus nephritis, with a SLEDAI score of 20. The treatment initiated included 250 mg of MP intravenously for three days, 0.75 g of MMF orally twice daily, 200 mg of HCQ twice daily, and 160 mg of Telitacicept subcutaneously once weekly.

After three weeks of treatment, the patient showed significant improvement and was discharged. She continues to attend regular outpatient follow-ups at our hospital. The latest review indicated normalization of CA-125 levels and resolution of ascites. The patient’s nephritis has transitioned to a chronic phase, and she is currently undergoing regular dialysis.

**Figure 3:**
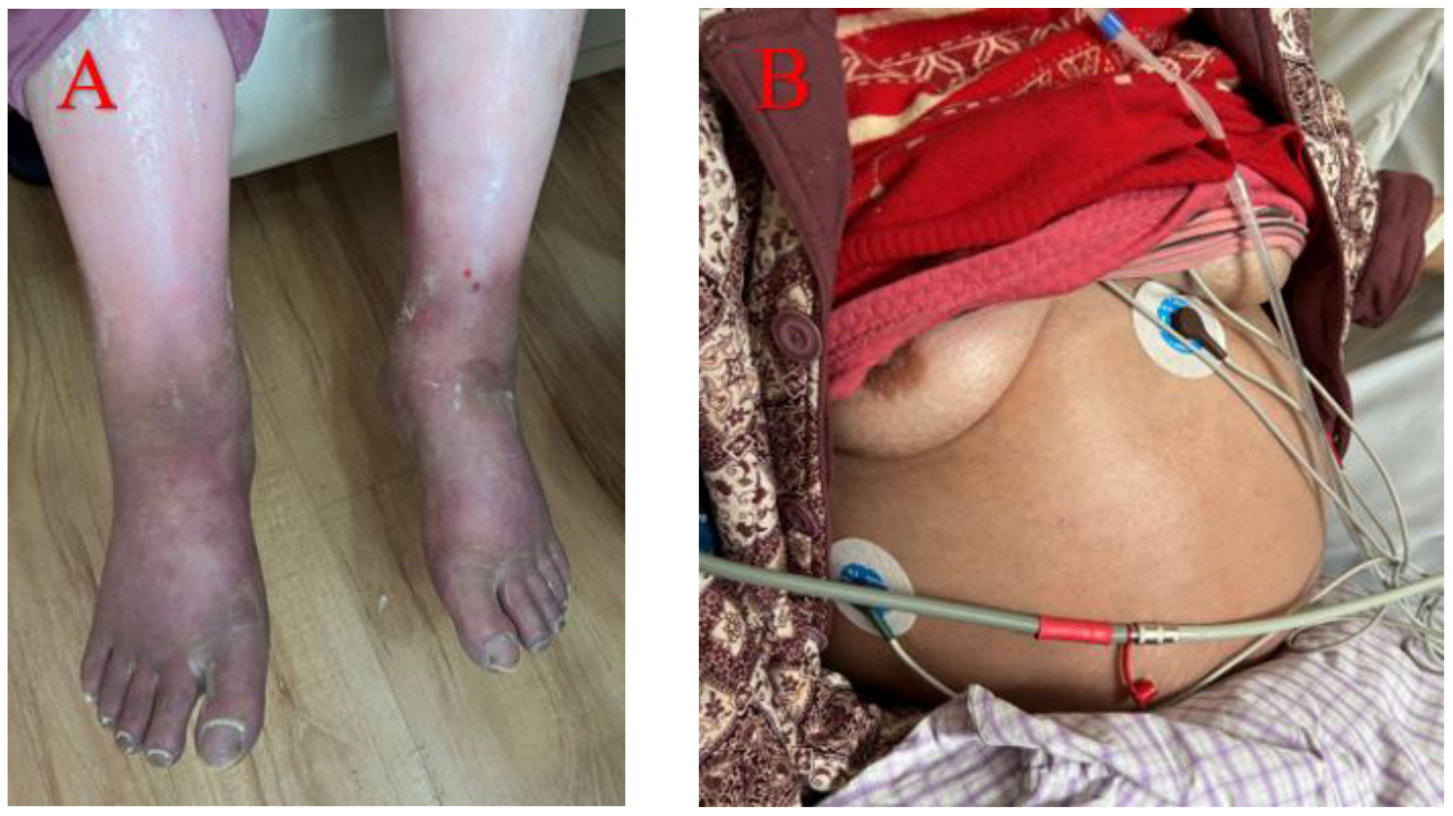
Five months before medical treatment, the patient developed depressed edema of both lower limbs **(A)**. Three months ago, she developed ascites of unknown origin **(B)**.

**Figure 4:**
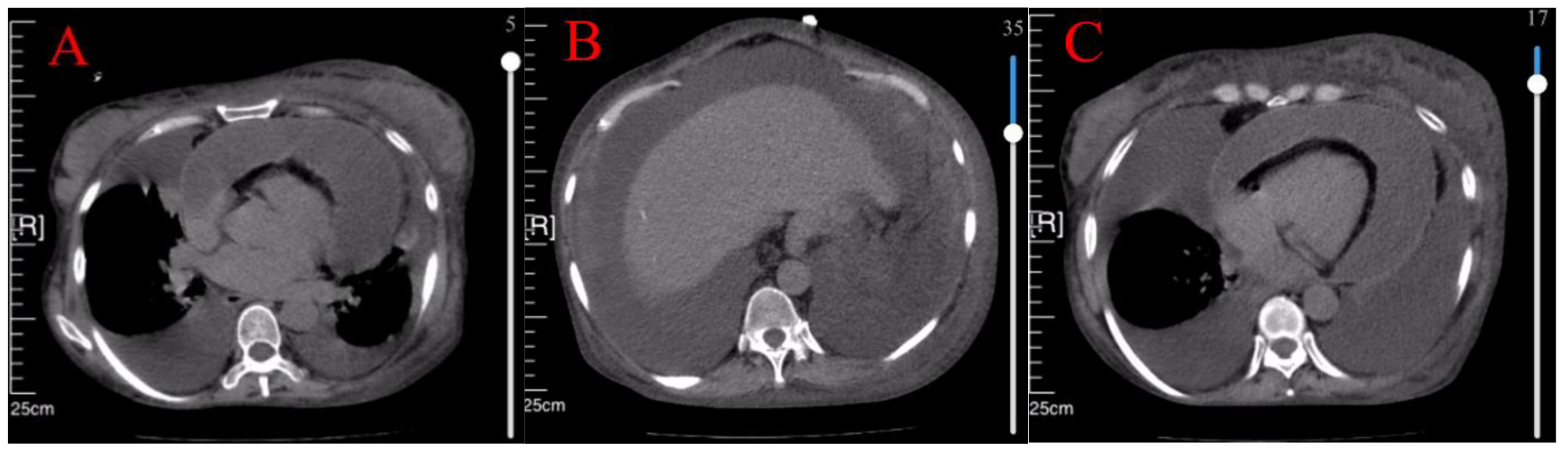
CT showed bilateral pleural effusion **(A)**, massive ascites **(B)**, and moderate pericardial effusion **(C)**.

Case 3 involved a 50-year-old woman diagnosed with Sjögren’s syndrome and depression approximately ten years earlier. Her adherence to the prescribed treatment and follow-up regimen was poor. She experienced unexplained lower extremity edema five months prior to seeking medical attention, which progressed to significant abdominal distension three months later (**Figure 3**). An initial assessment at an external hospital using CT imaging revealed bilateral pleural effusions, massive ascites and moderate pericardial effusion (**Figure 4A–C**). Serum CA-125 levels were recorded at 234.1 U/mL. Ascitic fluid CA-125 levels were notably high at 1013 U/mL following pericardial and abdominal paracentesis. The treatment administered included 120 mg of intravenous MP for three days and an unspecified dose of cyclophosphamide. However, her condition deteriorated, marked by acute dyspnea ten days post-treatment, necessitating a referral to our institution.

Upon reevaluation, significant bilateral pleural effusions were observed, leading to thoracentesis. Subsequent measurements showed a reduction in CA-125 levels in both serum (70 U/mL) and ascitic fluid (425 U/mL), compared to initial findings. PET-CT scans were performed to exclude malignancy. Tuberculosis was ruled out based on negative results for acid-fast bacilli smear and adenosine deaminase levels in pleural and ascitic fluids, as well as a negative whole-blood T-spot test. Rheumatoid antibody panels revealed an ANA titer of 1:320 with a nucleolar pattern, SSA and Ro-52 antibodies at 2+, negative anti-dsDNA and anti-SM antibodies, but positive ACA-IgG, β2-1-IgG, and Anti-C1q antibodies. Serum albumin was 27.5 g/L, with anemia (HGB 72 g/L). The patient exhibited significant hair loss, low complement levels (C3: 0.31 g/L; C4: 0.04 g/L), pleural and pericardial effusions, and renal involvement (serum creatinine: 101 µmol/L; 24-hour urinary total protein: 1.13 g/L). An SLEDAI score of 24 indicated active SLE with multiserositis. She was treated with 500 mg of intravenous MP for three days, followed by 200 mg of oral HCQ twice daily. After treatment, serum CA-125 levels decreased to 33.7 U/mL. Due to a concurrent COVID-19 infection, immunosuppressants were withheld, and intravenous MP was reduced to 40 mg daily. Given that the patient is currently experiencing highly active lupus and has tested positive for high titers of antiphospholipid antibodies, a concurrent diagnosis of antiphospholipid syndrome (APS) is considered. There is a risk of microvascular thrombosis, necessitating the addition of low molecular weight heparin for anticoagulation. Regrettably, the patient developed a secondary drainage infection during treatment, went into septic shock, and died of cardiac arrest.

## Discussion

This manuscript describes three cases of SLE where the initial presentation included significant pleural and abdominal ascites, alongside markedly elevated CA-125 levels. Notably, these patients had not previously been diagnosed with SLE. A distinctive feature of this series is the absence of a rheumatic disease history in the patient described in Case 1, in contrast to the patients in Cases 2 and 3, who had a documented history of Sjögren’s syndrome. Following the comprehensive exclusion of tumors, infections, and tuberculosis, the ascites and elevated CA-125 were determined to be manifestations of active SLE. Treatment with corticosteroids and immunosuppressants led to the alleviation of ascites symptoms and a significant reduction in CA-125 levels upon subsequent testing.

Serous inflammation, manifesting as pleuritis and/or pericarditis, occurs in approximately 16% of SLE patients. Lupus peritonitis with ascites, also referred to as lupus peritoneal effusion, is comparatively rare in SLE, representing approximately 8-12% of cases. It is even more uncommon as an initial presentation of the disease (3, 4).

Serum CA-125, a high-molecular-weight glycoprotein, is predominantly associated with pelvic malignancies, especially ovarian cancer. However, its expression in mesothelial cells, not exclusively in tumor cells, limits its diagnostic effectiveness for ovarian cancer due to reduced sensitivity and specificity. Elevated CA-125 levels can originate from various tissues, including the peritoneum, pleura, pericardium, fallopian tube epithelium, endometrium, cervix, lungs, and breast (5, 6). This elevation can occur under both normal and pathological conditions. Beyond malignant tumors, increased levels of CA-125 have been observed in conditions such as early pregnancy, peritonitis, menstruation, nephrotic syndrome, endometriosis, leukemia, congestive heart failure, liver cirrhosis, pulmonary vascular disease, rheumatoid arthritis, and tuberculosis (7).

When patients present with significant ascites and elevated serum and ascitic CA-125 levels, the differential diagnosis can be challenging for clinicians, often leading to considerations of pelvic tumors. Meigs’ syndrome, first delineated in 1937, is characterized by a triad that includes benign ovarian tumors (such as fibromas, thecomas, granulosa cell tumors, Brenner tumors, etc.), ascites, and pleural effusion (8). This syndrome typically resolves swiftly following the surgical removal of the tumor. Pseudo-Meigs’ syndrome encompasses similar clinical presentations resulting from conditions other than the tumor types originally specified in Meigs’ syndrome, often involving ovarian malignancies, such as primary or metastatic ovarian tumors, yolk sac tumors, ovarian cancers, leiomyomas, and hemangiomas (9).

The presentation of massive ascites, pleural effusion, and significantly elevated serum CA-125 levels in patients with SLE, in the absence of ovarian tumors, is referred to as pseudo-pseudo Meigs’ syndrome or Tjalma syndrome. Tjalma et al. documented the first case in 2005, involving an SLE patient with significant ascites and elevated CA-125 levels where gynecological surgery excluded malignancy (9). Another case, featured in The Lancet, detailed similar symptoms in an SLE patient, where treatment by internal medicine resolved the symptoms, establishing lupus activity rather than malignancy as the underlying cause. This condition has since been termed “pseudo-pseudo Meigs’ syndrome” (10).

In patients with SLE, the presence of serosal effusions is relatively common; however, the concurrent occurrence of significant ascites and markedly elevated CA-125 levels is rare. The identification of “pseudo-pseudo Meigs’ syndrome” serves as a crucial reminder for clinicians to consider differential diagnoses beyond ovarian tumors, thereby avoiding unnecessary surgical interventions. Nonetheless, pleural effusion in SLE lacks specificity and is often discussed in contexts mimicking Meigs’ syndrome. A more notable observation is the co-occurrence of substantial ascites and elevated CA-125 levels.

Although the exact mechanisms underlying significant ascites and elevated CA-125 in these cases remain unclear, current research suggests that in Meigs’ syndrome, tumor-induced peritoneal irritation leads to increased CA-125 production by peritoneal mesothelial cells (8, 9). In the context of SLE, disease activity initiates a pathological cascade including complement activation, immune complex deposition, vasculitis, and thrombotic damage. This process likely results in an increase in various cytokines, such as IL-1β and interferon-γ, which in turn may stimulate CA-125 expression in peritoneal cells (11). This intricate interplay highlights the need for a thorough evaluation to differentiate between underlying causes and to tailor appropriate management strategies.

Indeed, elevated serum CA-125 levels can be attributed to a variety of conditions, including nephrotic syndrome, lupus-associated protein-losing enteropathy (LUPLE), lupus peritonitis, and pelvic infections such as tuberculosis (12). The diagnosis of pseudo-pseudo Meigs’ syndrome (PPMS) requires the exclusion of lupus-related pathologies that cause polyserositis, such as lupus nephritis with nephrotic syndrome or lupus peritonitis. A review of the literature indicates that lupus patients with nephritis, peritonitis, LUPLE, or tuberculosis may also present with ascites or peritonitis (13). However, elevated CA-125 levels are rare and are typically only slightly above normal in a few cases. Diagnosing PPMS becomes more complex in scenarios where SLE patients present with other conditions that could cause ascites or mildly elevated CA-125 levels. For instance, Case 2 involved lupus nephritis, a known pathological factor for ascites, yet the significantly elevated CA-125 levels could not be solely explained by nephritis, nor could a PPMS diagnosis be ruled out. Cases 1 and 3 exhibit varying degrees of hypoalbuminemia, which is a common outcome of disease activity and consumption in SLE patients and is the initiating factor for pleural effusion and peritoneal effusion, but such a large amount of pleural effusion, ascites, and elevated CA-125 cannot be fully explained by low protein, preferring a PPMS diagnosis. To give priority to “monistic” explanations of disease progression, the diagnosis of PPMS should be considered first. Therefore, when diagnosing PPMS, focus should be placed on:

1. Significant ascites, pleural effusion, and elevated CA-125 levels that cannot be fully explained by other factors;

2. The presence of naive or exacerbated lupus activity;

3. Exclusion of intraperitoneal infectious diseases, benign and malignant ovarian tumors, including metastatic tumors;

4. Simultaneous resolution of ascites with significant reduction of CA-125 levels after SLE treatment is also of diagnostic significance.

We have compiled data from a global collection of 33 cases of PPMS, including this report, as detailed in **Table 1**. All reported patients were female, with ages ranging from 14 to 82 years, predominantly concentrated between 30 and 50 years. Given the gender ratio of approximately 1:10 in SLE patients (37) and the absence of male cases in PPMS reports, this prompts speculation regarding the role of female reproductive organs, such as the ovaries and adjacent adnexal structures, and their mesothelial cells in the high expression of CA-125. Additionally, except for two cases where complement levels were not reported, nearly all cases exhibited low complement levels, particularly low C3, highlighting the role of C3-binding immune complexes in the progression of PPMS. Contrary to other reports that consistently indicated significantly low C3, the C3 level in case 2 reported by Quintero-Munoz et al. was within the normal range (25). Upon reviewing this case, the patient’s final autopsy revealed an adnexal mass, pathologically identified as pseudomyxoma peritonei infiltrating the peritoneal wall. Therefore, it is questionable whether the clinical presentation was due to mucinous tumor infiltration rather than PPMS.

**Table 1:** Summary of clinical features of previously reported PPMS cases.

Year	Reporter	Gender	Age	Naïve SLE	Serum CA-125		Pericardial effusion	Renal impairment	Hypoproteinemia	Protei nuria	ANA	Ro/SSA	SM	dsDNA	Complement		Hematologic Involvement			APS	Fer	Serum Calcium	Treatment	Outcome
					initial	post-treatment									C3	C4	WBC	HGB	PLT					
2005	Tjalma (9)	Female	38	+	887	normal		-										↓	↓	+			MP+AZA	Remission
2005	Schmitt (10)	Female	33		1239	12		-	+	+	+			+	↓	↓	↓	↓	↓	+			MP + MMF + HCQ	Remission
2008	Ural (14)	Female	38		1229	normal				-				+	↓	normal							MP+HCQ	Remission
2011	Bes (15)	Female	47		233	9	+				+	+		+	↓	↓	↓	↓					MP+HCQ	Remission
2012	Dalvi (16)	Female	56	+	70				+	+				+	↓	↓							MP+MMF	Remission
2013	Bes (17)	Female	42		91		+		+	+	+			+	↓	normal			↓		↑	↓	MP+CYC/AZA	Remission
2013	Lee (11)	Female	29		345	12	+	+		-	+			+	↓	↓	↓	↓	↓	+	↑		MP+HCQ	Remission
		Female	54	+	345		+	+	+	-					↓	↓		↓			↑		MP+CYC/MMF	Remission
2016	Cheah (18)	Female	34		1614	normal	−		+		+			−				↓					MP+HCQ	Remission
2016	McVorran (19)	Female	40		307		+	-			+	+		+	↓	↓					−		MP	Remission
2018	Zampeli (20)	Female	40	+	85		+				+		+	+	↓	↓							MP+MMF/CYC	Remission
2019	Torres (7)	Female	14		59	23		+	+			+			↓	↓		↓	↓				MP+CYC/MMF+RTX	Remission
2019	Ahmed (21)	Female	44	+	227		−	-	+		+			−	↓	↓					−		MP+AZA	Remission
2019	Gao (13)	Female	44		↑			+	+	+	+			+	↓	↓	↓	↓					MP+HCQ+LEF	Remission
2019	Li (22)	Female	24	+	949		+	-	+	-	+	+	−	−	↓	↓	↓		↓		−		MP+MMF	Remission
2019	Tansir (23)	Female	22		2025		+		+	+				+	↓	↓		↓	↓	+	↑		MP+HCQ+CYC/AZA	Remission
2019	Awad (24)	Female	43	+	80			+	−									↓					MP+MMF+HCQ	Remission
2021	Quintero-Munoz (25)	Female	33	+	187		−	-	+	+			+	+	↓	↓				+			MP+HCQ+MMF	Death
		Female	40	+	54		+				+			+	normal	normal		↓		+			MP+HCQ+AZA	Death
2021	Meena (26)	Female	23	+	231	12		-	+	-	+	+	+	+	↓	normal		↓	↓	+	↑		MP+AZA+HCQ	Remission
2022	Horino (27)	Female	51		763	16																	DEX	Remission
			53		768				+		+		+		↓	↓							MP+CTX+HCQ+ IVIG	Remission
2022	Wang (28)	Female	23		1685	22	−		+	-	+	+			↓	↓		↓	↓				MP+HCQ	Remission
2022	Karadeniz (29)	Female	33		476		+	+		+	+	+		+	↓	↓					↑		MP+MMF+HCQ	Remission
2022	Chao (30)	Female	82		323	11		+			+		+	−	↓	↓		↓	↓				MP+HCQ	Remission
2022	Hammadi (31)	Female	41		121			-	−	-	+				↓	normal		↓					MP+MMF+HCQ	Remission
2022	Martins (32)	Female	48		60		+	+	+	+	+			+	↓	↓	↓	↓	↓		↑		MP+HCQ+CYC/MMF	Remission
2023	Tan (33)	Female	32		2767	42		+		-	+		+	+				↓				↑	MP+MMF+HCQ	Remission
2023	Dang (34)	Female	74	+	155		−	-		-	+												MP+MMF	unknown
2023	He (35)	Female	47		962	normal		+	−	-	+			−	↓	normal					↑		MP	Remission
2024	Deniz (36)	Female	33		71	68	+	+	+	+	+	+	+	+	↓	↓	↓	↓					MP+MMF+HCQ	Remission
2024	Current Cases	Female	28		1225	99	+	-	+	-	+	+		−	↓	↓							MP+MMF+HCQ +Telitacicept	Remission
		Female	37		186	10.3	+	+	+	+	+	+		+	↓	↓		↓	↓		↑		MP+MMF+HCQ +Telitacicept	Remission
		Female	50		234	34	+	+	+	+	+	+	−	−	↓	↓		↓		+	↑		MP+HCQ+ IVIG	Death

SLE, Systemic lupus erythematosus; CA-125, Cancer Antigen 125; ANA, Antinuclear antibodies; SM, Anti-Smith Antibody; dsDNA, Anti-double-stranded DNA antibodies; WBC, White Blood Cell; HGB, Hemoglobin; PLT, Platelet; APS, Antiphospholipid syndrome; Fer, Ferritin; MP, Methylprednisolone; AZA, Azathioprine; MMF, Mycophenolate mofetil; HCQ, Hydroxychloroquine; CYC, Cyclophosphamide; RTX, Rituximab; LEF, Leflunomide; DEX, Dexamethasone; IVIG, Intravenous Immunoglobulin.

-, negative; +, positive; ↑, elevated; ↓, decreased.

In the clinical presentation of 33 cases, pericardial effusion was reported in 15 cases (45%), impaired renal function in 13 cases (39%), hypoproteinemia in 19 cases (58%), positive urinary protein in 10 cases (30%), and positive antinuclear antibodies (ANA) in 25 cases (76%). Additionally, SM antibodies were positive in 7 cases (21%), dsDNA in 18 cases (55%), Ro/SSA in 11 cases (33%), and hematological system involvement was reported in 22 cases (67%). This hematological involvement included leukopenia in 7 cases (21%), anemia in 20 cases (61%), and thrombocytopenia in 12 cases (36%). Eight cases were reported to have associated APS (24%). Due to missing data in some case reports, these percentages might slightly underestimate the actual figures but still provide a useful reference for clinical consideration.

Furthermore, research indicates a correlation between hyperferritinemia and heightened disease activity in patients with PPMS. Among the cases reporting ferritin levels, 10 exhibited hyperferritinemia, whereas 3 cases reported normal ferritin levels. Ferritin is the main source of activated macrophages under inflammation, representing the inflammatory response, and the increase of Fer can be observed in a variety of inflammatory diseases (including autoimmune and infectious) (38), so its specificity in this disease is controversial. However, the degree of its elevation reflects the severity of inflammatory activity to a certain extent, and can still provide some reference value for clinical decision-making.

In addition, studies have reported instances of hypercalcemia in patients with PPMS, suggesting that hypercalcemia might serve as a potential marker of SLE activity, associated with autoantibody production and pro-inflammatory factors. It may even manifest as an initial symptom of SLE (39). However, in SLE patients, factors such as avoidance of UV exposure, potential renal or gastrointestinal complications impairing calcium absorption, and calcium loss due to prolonged hormone therapy are prevalent (2). Consequently, hypocalcemia is more commonly observed, as documented by Bes, C., et al. (17). In the three cases we reported, blood calcium levels were within normal ranges. Therefore, whether hypercalcemia may represent a specific phenotype in PPMS patients warrants further investigation and additional case reports for confirmation.

In managing PPMS, the use of immunosuppressants and corticosteroids to reduce SLE activity is a standard and widely accepted approach, with nearly all cases reporting the efficacy of corticosteroids. Following treatment, almost all cases reported alleviation of clinical symptoms, and nearly half of the cases reported a significant reduction in CA-125 levels. Notably, of all reported cases, only in the case reported by Horino et al. (27), the patient experienced lupus activity with abundant ascites for the first time, and the symptoms resolved after treatment. However, after 2 years, the patient’s lupus disease became active again, with the reappearance of massive ascites. Among the reported cases, eight were associated with definitive APS. The binding of antiphospholipid antibodies (aPL) to receptors on vascular endothelial cells results in endothelial damage, increased vascular permeability, and enhanced fluid extravasation. This interaction triggers hypercoagulability and the formation of microthrombi, leading to organ ischemia (40). In the three reported fatalities, all patients were diagnosed with APS. Autopsy findings reported by Elias Quintero-Muñoz et al. (25) confirmed extensive microthrombosis, which likely contributed directly to the fatalities. These observations underscore the necessity for heightened clinical vigilance and the timely initiation of anticoagulant therapy in the patients of PPMS combined with APS.

Furthermore, some studies have reported elevated cytokine levels in PPMS patients, including serum levels of IL-2, IL-4, IL-5, IL-10, IL-17, and IL-12p70, as well as pleural levels of IFN-γ, IL-6, and IL-8, suggesting that biologic therapies targeting these inflammatory cytokines might offer potential benefits. Treatment options could include therapies inhibiting the IL-23/IL-17 axis or antibodies targeting the Janus kinase/Signal Transducer and Activator of Transcription (Jak/Stat) pathway, representing a new direction in the treatment of PPMS (41). In Cases 1 and 2 we report, the patients received treatment with the dual-targeted B-cell biologic agent, telitacicept. The mechanism of action of telitacicept includes the inhibition of B-cell maturation, differentiation, antibody secretion by plasma cells, and the production of autoantibodies (42–44). Its efficacy and safety have been demonstrated in SLE (45, 46). The adjunctive use of telitacicept in standard therapy not only helps in rapidly reducing the dosage of corticosteroids, thereby minimizing the adverse effects associated with long-term use of high doses of corticosteroids, but also effectively promotes clinical remission.

These observations underscore the complexity of PPMS, indicating that an integrated approach addressing both the underlying SLE activity and the distinct pathological features specific to PPMS is likely required for effective management. Immune complex-mediated CA-125 secretion by mesothelial cells may play a key role in the pathogenesis of PPMS. In the future, testing for autoantibody profiles in blood and bodily fluids, and identifying correlations between specific autoantibodies and CA-125 secretion could provide new insights into the mechanisms underlying PPMS. Employing targeted therapies that modulate the unique inflammatory profile of PPMS may open new therapeutic pathways, potentially enhancing outcomes for patients with this uncommon manifestation of SLE.

Pseudo-pseudo Meigs’ syndrome is a distinct phenotype of SLE, initially characterized by pronounced pleural effusion, ascites, and significantly elevated CA-125 levels. It is imperative for clinicians to recognize the characteristics of this condition to distinguish it effectively from Meigs’ syndrome and pseudo-Meigs’ syndrome, thus preventing misdiagnoses as ovarian tumors and averting unnecessary surgical interventions.

Due to the relatively limited number of cases reported thus far, the underlying mechanisms of PPMS remain incompletely understood. Nevertheless, it is currently acknowledged that widespread activation of the complement system, deposition of immune complexes, vasculitis, and thrombotic damage, which stimulate peritoneal mesothelial cells to produce ascites and highly express CA-125, comprise the pathological basis of this condition. In managing PPMS, immunosuppressants and corticosteroids have been demonstrated to be effective. Additionally, targeting B cells, Tcells or inflammatory cytokines may also be a promising approach.

## Data availability statement

The original contributions presented in the study are included in the article material. Further inquiries can be directed to the corresponding author, Bailing Tian (tianbailing2010@163.com).

## Ethics statement

Written informed consent was obtained from the patient for the publication of this case report.

## Author contributions

QL: Writing – original draft. BT: Resources, Writing – review & editing.
